# Blending Martial Arts and Yoga for Health: From the Last Samurai to the First Odaka Yoga Warrior

**DOI:** 10.3389/fsoc.2020.597845

**Published:** 2020-11-16

**Authors:** Matteo Di Placido

**Affiliations:** Department of Sociology and Social Research, University of Milan-Bicocca, Milan, Italy

**Keywords:** martial arts, yoga, Odaka yoga, health, subtle body

## Abstract

This paper introduces the case study of Odaka Yoga, an innovative style of postural yoga blended with martial arts elements which emphasizes the importance of practitioners' health and processes of self-transformation as pivotal to the school's ethos. More specifically, the paper explores how Odaka Yoga's philosophical backdrops and practical repertoire, composed by a mixture of “exotic” resources such as Bushido, zen, yoga, and a constant reference to the ocean waves and biomechanics, constitute a very specific vision of *health* at the intersection of Western science and esoteric knowledge. Theoretically, the paper borrows from Jennings' theory of *martial creation* and enriches it with some of the central analytical tools proposed by theorists such as Bourdieu and Foucault. Methodologically, it relies on a *multimodal approach* including *discursive analysis* of the school's promotional materials, *interviews* with the founders and other key teachers, and *observant participation* of practitioners' apprenticeship processes. More Specifically, this paper discusses the birth of Odaka Yoga as occurring at the intersection of Asian martial arts and yoga, as well as the founders' biographical trajectories from the world of competitive martial arts and fitness, to yoga; it then turns to an examination of Odaka Yoga's conception of health as a mixture of the Western biomedical model and the subtle body model of Asian traditions such as yoga and martial arts. It argues that the conception of health promoted by this school gives rise to the Odaka Yoga Warrior, the ideal-typical practitioner whose body is simultaneously exposed to the medical gaze and its imperatives of control, knowledge, and manipulation; while it also deifies it, as it is animated by the elusive flows of energy (*qi or prana*) that prolonged practice aims to master. The paper concludes with a reflection on hybrid conceptions of health and the ubiquitous role of health discourses and narratives across sociocultural domains.

## Introduction

Martial arts, defined as the combat arts that have developed—and continue to develop—across geographies and cultural contexts all over the globe, are, in the public and popular imagination, tightly connected with spectacular flying kicks, violence, and superhuman powers. Nevertheless, a significant number of martial arts and “martial activities” (Martínková and Parry, 2016) more in general (e.g., Aikido, Karate, Yoga, Taijiquan, Wing Chun Kung and Qigong, among others), are also substantially dedicated to the maintenance, improvement, and restoration of the practitioner's health from both a philosophical/discursive standpoint, and a practical/experiential level of consideration. Social science literature, however, especially those studies produced within the disciplinary interests of “martial arts and combat sports (MACS)” (e.g., Farrer and Whalen-Bridge, 2011; Sánchez-García and Spencer, 2013; Channon and Jennings, 2014), and “martial arts studies” (Bowman, 2014, 2015, 2017, 2018), have so far contributed to provide a picture of martial arts as first and foremost a fearsome activity dominated by action, physical confrontation and violence. This literature has extensively inquired into the history, developments and deployments of many martial arts (e.g., Green and Svinth, 2003) and their deconstruction (e.g., Bowman, 2019a), focusing especially on their “culture of combats” (e.g., Sánchez-García and Spencer, 2013; Brown et al., 2019), pedagogical environments, processes of apprenticeship, and knowledge transmission (e.g., Wacquant, 2004; Brown, 2005, 2011; Downey, 2005, 2008; Spencer, 2009, 2014; Brown and Jennings, 2011; Downey et al., 2015; Jennings et al., 2020), embodiment and sensuous involvement (e.g., Stephens and Delamont, 2006; Samudra, 2008; Farrer and Whalen-Bridge, 2011; Spencer, 2011, 2012; Jennings, 2013; Channon and Jennings, 2014; Southwood and Delamont, 2018; Telles et al., 2018), religious and spiritual bearings (e.g., Maliszewski, 1996; Brown et al., 2009, 2014; Jennings et al., 2010; Brown, 2013; Tuckett, 2016; Pedrini, 2020), and media representations (e.g., Brown et al., 2008; Jakubowska et al., 2016; Yip, 2017; Bowman, 2019b,c,d, 2020a,b; Trausch, 2019). Moreover, as this body of work increases, specializes and further develops, also its attention to conceptual clarity and theoretical developments intensifies, with the consequent introduction of a host of new concepts and theoretical perspectives (e.g., Brown and Jennings, 2013; Sánchez-García and Spencer, 2013; Cynarski and Skowron, 2014; Martínková and Parry, 2016; Bowman, 2017; Cynarski, 2017, 2019a; Jenninings, 2019; Pedrini et al., 2019) oriented to the creation, maintenance, and re-invention of the disciplinary boundaries of martial arts and combat sports and martial arts studies and to their legitimacy as autonomous fields of study. However, as rightly underlined in the journal's description of this special issue on ‘*Martial Arts, Health and Society*’, “[h]ow martial activities might be health-giving, dangerous or healing, therapeutic, and rehabilitative activities connected to ideas on the body and medicine remain largely unaddressed”^1^. I contend that this lack of research on health and related issues in the martial arts is largely due to their popular representations and academic renditions as first and foremost arts devoted to combat. Emerging categories such as “martial activities” (Martínková and Parry, 2016) and “martialité” (martiality in English) (De Grave, 2019), on the other hand, show the capacity to bridge the study of martial arts with the study of an array of other physical and non-physical practices oriented to the disciplined cultivation of the practitioner's mind, body and soul, and because of this, are useful in accounting for how health is theorized, transmitted, and cultivated in specific martial arts and martial activities. This paper, therefore, together with those studies that have already begun to competently inquiry into the relationships between martial activities and health in contemporary societies (e.g., Alter, 2004; Brown and Leledaki, 2005, 2010; Burke et al., 2007; Newcombe, 2007, 2019; Croom, 2014; Fong, 2014; Jain, 2014, 2020; Jennings, 2014, 2017; Leledaki, 2014; Cynarski and Sieber, 2015; Markula and Chikinda, 2017; Smith and Atencio, 2017; Cynarski, 2018, 2019b), attempts to be a contribution in this direction. More specifically, I contribute to the literature with an ethnographic and discursive exploration of Odaka Yoga, an innovative style of postural yoga blended with martial arts elements, which emphasizes the importance of practitioners' health and processes of self-transformation as pivotal to the school's ethos. In this way, I explore how Odaka Yoga's philosophical backdrops and practical repertoire, composed by a mixture of “exotic” resources such as Bushido^2^, zen, yoga, and a constant reference to the ocean waves and biomechanics, constitute a very specific vision of health at the intersection of Western science and esoteric knowledge. It is important to mention, in fact, that this school presents itself as an illustrative case of the changing nature of martial arts, and modern forms of yoga and their adaptation to different sociocultural environments. More specifically, the eclectic philosophical and discursive backdrops of Odaka Yoga are a poignant example of the legitimizing and differentiating strategies that contemporary yoga brands follow in the attempt to conquer a share of the already saturated and highly commodified “spiritual marketplace” (Roof, 1999). Here, Odaka Yoga's promises for health and self-transformation are central to its image, marketization, and popularity.

This paper is structured as follows: first, I briefly discuss the theoretical and methodological backbones of the paper via a reading of Jennings' (2019) “theory of martial creation” and a “multimodal approach” (Brown and Leledaki, 2010) inclusive of discursive analysis of the school's promotional materials, interviews to the founders, and other key teachers and “observant participation” (Wacquant, 2004, 2015) of practitioners' apprenticeship processes; second, I reconstruct the birth of Odaka Yoga at the intersection of Asian martial arts and yoga, presenting the biographical trajectories of the founders Roberto Milletti and Francesca Cassia, from the world of competitive martial arts and fitness, to yoga; third, I delve deeper into an exploration of Odaka Yoga's conception of health, as chiefly the mixture of a Western biomedical view with the subtle body model of Asian traditions. In so doing, I argue that the conception of health promoted by this school gives rise to the Odaka Yoga Warrior, the ideal-typical practitioner whose body is simultaneously exposed to the “medical gaze” (Foucault, 1977) and its imperatives of control, knowledge and manipulation; while it also deifies it, as it is animated by the elusive flows of energy (*qi or prana*) that prolonged practice aims to master; fourth, the paper concludes with a reflection on hybrid conceptions of health and the ubiquitous role of health discourses and narratives across sociocultural domains.

## Theoretical and Methodological Sketches

### Jennings' Theory of Martial Creation and Other Remarks

Jennings' (2019) “theory of martial creation” emerges at the intersection of Mills's (1959) “sociological imagination” and Shilling (2008) call for a more pragmatist orientation to the study of the body. The former, a reaction to the structural-functionalism of Talcott Parsons' sociology, is primarily concerned with directing scholars' attention to the manners in which macro-sociocultural processes and historical events intertwine with—and could be studied through—individual biographies; the latter, distancing itself from what it perceives to be the limits of “the dominant traditions in sociology” (Shilling, 2008:3), rehabilitates the pragmatist notion of “habit” (Dewey, 1922) and focuses on moments of crises that stimulate social actors' creativity and actions oriented to social change. As Jennings' (2019:62) comments, “[t]aken together, these provide a powerful framework for understanding how and why a person might create a new martial arts system,” and I would like to add—in line with the purpose of this paper—also the specific health philosophies and conceptions of health that are informing a given martial arts system.

Jennings develops this innovative framework through a close exploration of three traumatic moments in Bruce Lee's life: first, Lee's attempts to adapt and change his acquired knowledge, mastery of wing chun and training regimes after his geographical separation from his teacher Ip Man; second, Lee's physical crises leading him to develop his training methods in search of a better fitness regime; and third, a severe impairing back injury which forced Lee to adapt *à nouveau* his “martial habitus” (Brown and Jennings, 2013). In a nutshell, Jennings' theory links Lee's personal and biographical elements with social and historical dimensions like US racism, Chinese ethnocentric nationalism and traditional kinship relationships in Chinese martial arts to show how he arrived, through a series of crises, serendipitous happenings, and creative resolves, to the foundation of a new system of martial arts he called *jeet kune do*.

The theory of martial creation proposes “six precise dimensions” that constitute the backbones of the process of creating a new martial art. They are (Jennings, 2019:65, emphasis in original):

1. Founders must have a background as *practitioners* in one or more martial art(s).
2. They must achieve a level of *competence, confidence*, and *charisma* in order to gather a following.
3. Yet, they will *not* be the top students, official gatekeepers, or lineage holders of their original system.
4. They must identify a *problem* or face a personal, political, or social *crisis* that aggrieves them.
5. They will then devise a *solution* through a revised fighting, human development, and training system.
6. Their passing (whether expected or unexpected) can create added *chaos*, thus fuelling the cycle of *creativity* among future generations of practitioners.

In Jennings' (2019:65) words, “these are some of the crucial dimensions that founders of martial arts can be seen to have possessed and hence constitute dimensions that will be present for all who create fighting systems.” As we will see in the course of this paper, the founder's biographies could be equally mobilized in the light of these six points to account for the birth and development of Odaka Yoga. However, contrary to Jennings' focus on the creation of martial arts as fighting systems, I contend that his theory could be also fruitfully adopted and adapted to discuss the health philosophies underpinning specific martial activities. More precisely, as I will unpack in some details in the analytical sections of this paper, Jennings' theory is instrumental in tracing the processes of appropriation, development and re-invention of specific health philosophies and hybrid conceptions of health emerging at the intersection of the Odaka Yoga's founders biographical transformations (epitomized by moments of crises and re-definition of their own martial habitus) and social change (represented by the broader processes of transformation characterizing a particular historical moment and the consequent processes of re-definition of the internal logics of specific fields of action).

The theory of martial creation has its strengths and its weaknesses. Among its main strengths figure: first, its ability to overcome the oversimplistic approach that privileges the production of stringent definitions and taxonomies rather than theories—and I would add theorizing—in both the field of martial arts studies (Bowman, 2017) and in the social sciences more in general; second, Jennings' theory is not merely instrumental in theorizing about the birth, development, adaptation, and change of a number of martial arts, but it could also be applied to the study of other practices and organizational contexts centered around a charismatic leader, such as different yoga schools, New Religious Movements and other spiritual and religious organizations. For instance, this theory could shed light on how contemporary gurus shape their teachings at the intersection of tradition and innovation, or again, on the manners in which New Religious Movements syncretically construct, justify, and transmit their religious and spiritual worldviews; third, it could be used side by side a number of qualitative research methods, such as “ethnographic and media as well as biographical, historical and sociological research methods,” as Jennings' (2019:69) himself acknowledges; fourth, it could also be mobilized across a plethora of theoretical frameworks, being itself further developed by a careful integration of other conceptual tools.

As I intend to show in the remaining of this section, Jennings' theory of martial creation could be further developed through a more thorough discussion of Bourdieu concepts of “habitus” (e.g., 1977; 1984; 1990) and “field” (e.g., 1971; 1983; 1996), and Foucault (e.g., 1986; 2005) seminal analysis of “epimeleia heautou” (literally care of oneself). In fact, I believe that among the weaknesses of this theory there is the reproduction of Shilling (2005, 2008) misleading critique of Bourdieu's concept of habitus as substantially deterministic. As Shilling (2008:3, emphasis in original) had expressively argued:

The problem with this [habitus] is that embodied action appears predetermined—it both echoes and replicates existing structures—leaving those who operationalise Bourdieu's work in their research employing strategies to modify its reproductive logic.

However, the concept of habitus, defined as “systems of durable, transposable *dispositions*, structured structures predisposed to function as structuring structures” (Bourdieu, 1977:72, emphasis in original), is an ingenious account of social action capable of bridging the structure-agency gap while accounting for social change and not merely social reproduction. More specifically, the concept of habitus postulates the manners in which the objective structures of a given social order (e.g., language, culture, economy, laws, and so on) deposit within individuals' bodies as “schemes of perception, thought, and action” (Bourdieu, 1977:90), which in turn influence the forms and functioning of these very objective structures and individuals' ability to navigate them. As underlined by Bourdieu (2005:45, emphasis in original), “[t]he habitus is not a fate, not a destiny…the model of the circle, the vicious cycle of structures producing habitus which reproduces structures *ad infinitum* is a product of commentators.” As Bourdieu (2005:46) further comments “habitus is never a mere principle of repetition… It is a principle of invention, a principle of improvisation. The habitus generates inventions and improvisations but within limits.” In this light, the diffuse reading of the habitus as intrinsically deterministic (e.g., Shilling, 2005, 2008; Yang, 2014) appears, to say the least, only partial, and tangential to Bourdieu's formulation of the concept.

Moreover, next to a dismissal of habitus (more radical in Shilling than in Jennings), what I contend is missing from Shilling's as much as from Jennings's respective theoretical projects, is the recognition that Bourdieu's concept of “field” (e.g., Bourdieu, 1971, 1983, 1996) is central to fully understand—and in turn mobilize—his concept of habitus. As succinctly suggested by Bourdieu and Wacquant (1992:97), “a field may be defined as a network, or a configuration, of objective relations between positions” which shape—and is, respectively, shaped—by social actors' habitus. Bourdieu discusses at length the inextricable relationships between “positions” in a given field, determined by the amount and types of “capital” (e.g., Bourdieu, 1986) held by social actors, and “dispositions.” The former, “contrary to a Marxist understanding is defined as a form of accumulated labor composed of both material and symbolic resources” (Di Placido, 2018:7), while the latter are the constituting elements of the habitus (e.g., Bourdieu, 1983). For instance, in his seminal articles ‘*Genèse et Structure du Champ Religieux*’ (1971) and ‘*The Field of Cultural Production*’ (1983), and in his books ‘*Homo Academicus*’ (1988) and ‘*The Rules of Art: Genesis and Structure of the Literary Field*’ (1996), Bourdieu applies his concepts of habitus, capital, and field, to discuss the genesis, internal articulation, and struggles over different positions, in the religious, cultural academic, and literary fields. Here, as elsewhere, Bourdieu meticulously traces the manners in which the field and the habitus—of course always determined by the types and amounts of capital held by social actors—shape one another. More specifically, Bourdieu and Wacquant (1992:127, emphasis in original) argue that:

The relation between habitus and field operates in two ways. On one side, it is a relation of *conditioning:* the field structures the habitus, which is the product of the embodiment of the immanent necessity of a field (or of a set of intersecting fields, the extent of their intersection or discrepancy being at the root of a divided or even torn habitus). On the other side, it is a relation of knowledge or *cognitive construction*. Habitus contributes to constituting the field as a meaningful world, a world endowed with sense and value, in which it is worth investing one's energy.

In this sense, I argue that Jennings' theory could be further strengthened if equipped with the theoretical tools to competently inquire into these relationships of conditioning and of knowledge between the field of martial arts and the “habit(us)”^3^ (Jennings, 2019:64) of martial artists.

Another central element of Bourdieu's concept of field is the relationships of “homology” (e.g., Bourdieu, 1983) that he identifies between social actors' positioning in a given field and in the broader field of power. According to this principle, although each field (e.g., religious, cultural, academic, literary, sporting, martial arts, and so on) is first and foremost an independent and autonomous field from the broader social field, social actors' capital, and habitus tend to shape it, reproducing within it—at least partially—the same schemata of positions and relationships of domination and submission that characterize the general field of power. Therefore, as I will preliminary argue in the analytical part of this paper, when studying the genesis of a new martial activity such as Odaka Yoga, it is important to not only understand the founders' habitus and their positioning within the fields of martial arts, fitness, and well-ness industry, but also their positioning within the broader field of power. This extended focus equips us to more competently inquire into the links between individuals' biographies and historical events, as well as the manners in which the habitus is challenged by unexpected changes and reconfiguration in the field in which social actors are positioned. This, in turn, stimulates social actors' adaptation, drive to change, and creative resolve.

Additionally, I would like to discuss Foucault's (1986, 2005) analysis of “epimeleia heautou” as instrumental to further develop and enrich Jennings' theory of martial creation. With this expression, Foucault addresses all those processes and practices of constitution of the individual as an ethical subject of reason that he studied in relation to ancient Greece, Hellenism and the early Roman Empire. However, leaving aside the differences between the various systems of care of the self-put forward by Platonists, Epicureans, Stoics and early Christians, Foucault's framework of care of the self proposes: first, a general moral framework, or as he says, “…an attitude toward the self, others, and the world” (Foucault, 2005:10) represented by the doctrines and the principles of the philosophical schools in question (e.g., Platonism etc.,); second, an experience of conversion “…from the outside, from others and the world etc., toward “one self” (Foucault, 2005:11), whereby individuals are encouraged to cultivate a specific introspective relationship to “what we think and what takes place in our thought” (ibidem); and third, those specific practices, or “technologies of the self,” “exercised on the self by the self” whose primary aim is to allow the individual to “take responsibility for oneself and by which one changes, purifies, transforms, and transfigures oneself” (ibidem). As I will show in the analytical part of this paper, these three elements of Foucault's care of the self, that is, the moral universe of a specific martial activity, the experiences of conversion that it fosters, and its practices, or technologies of the self, can be fruitfully mobilized to study the birth, development and evolution of different martial activities alongside Jennings's theory of martial creation. More specifically, the first element of Foucault's formulation could be used to understand the specific worldview, desire, need, and/or possibility to change that martial arts' founders possess; the second element of conversion could be read as an epiphany, or a moment of crises, that further strengthens the martial artist's resolve to change, reinforcing his or her conviction in a new moral universe and/or creative resolve; and finally, the third element, technologies of the self, could be understood as the practical means that grant martial artists the opportunity to work on themselves, transforming and changing their habitus in the process of creating a new martial art.

To conclude, Jennings' theory of martial creation, enriched by a closer exploration of Bourdieu's and Foucault's conceptual tools, becomes particularly suitable to the study of those health philosophies and conceptions of health that are simultaneously: internalized, transformed and reproduced—in practice—through the cultivation of a specific habitus and its process of transformations, adaptation, and change to new biographical and historical events; determined by the internal logics of certain fields, organized around the centrality of the construct of health (e.g., the well-ness industry); and finally, advocated for as an integral part of a specific practice of care of the self-instrumental to martial artists' processes of self-cultivation.

### A Multimodal Approach

A “multimodal approach” to qualitative research considers simultaneously the macro (e.g., large scale changes in society), the meso (specific fields, organizational, and social contexts) and micro (practitioners' experiences and biographies) levels of society (e.g., Brown and Leledaki, 2010; Jennings, 2014). As I apply it to my study of Odaka Yoga, a multimodal approach is constituted by a number of different research methods, such as *discursive analysis* of Odaka Yoga's promotional materials, *biographical interviews* of founders and key teachers, and to a lesser degree, *observant participation* of practitioners' apprenticeship processes.

To put it briefly, I rely on discursive analysis as the study of “discourse,” understood with Foucault (1972:49) as a historically contingent social system that produces knowledge and meaning through “practices that systematically form the objects of which they speak.” In this light, discourse transcends an understanding of mere textual and/or spoken words. More specifically, “.discourses are composed of signs; but what they do is more than use these signs to designate things. It is this *more* that renders them irreducible to the language (*langue*) and to speech” (Foucault, 1972:49, emphasis in original). Discourses are inherently related to a variety of social practices, to the consequent constitution of their object of analysis as a “real entity,” and being historically contingent, are always structurally related to the broader structures of knowledge that characterize the historical period they arise in, that is its “episteme”^4^. Therefore, through a discursive analysis of Odaka Yoga's promotional materials, I aim to bring to the forth the social and discursive construction of the Asian traditions and systems of knowledge the school mobilizes, to produce health-based, therapeutic, and self-transformative resources.

Continuing, I discuss practitioners' narratives and experiences via “biographical interviews.” More specifically, the biographical interview method here used situates itself at the intersection of a “contextual approach” to biographical interviews, that is primarily concerned with the “realism” of the narrative gathered; and an “interpretative approach” which poses its emphasis on the socially constructed nature of the narratives and their fictional character (e.g., Roberts, 2002; Nilsen and Brannen, 2010; Gomensoro and Burgos Paredes, 2017:157). As the “contextual approach” it is concerned with the “lived life,” the “told story,” and the “self-interpretation” of social actors' life, although it is critical of its realist epistemology and of any naïve reading of social actors' narratives of self-determination. While in agreement with the “interpretative approach” it recognizes the importance of the researchers' own interpretation of the narratives gathered, their socially constructed nature, and their fictional character.

Theorizing at the intersection of the social actors' point of view and the broader sociocultural context in which they are located, I follow Mills's (1959) interpretative sociology and his invitation to study society through a close scrutiny of social actors' biographical experiences, that is, I inquire into the manners in which the discourses explored through my discursive analysis are then embodied, internalized, and reproduced by the social actors' narratives. Importantly, in this paper, I only use two out of the twenty-five biographical interviews conducted within my larger sociological project on modern forms of yoga, exclusively presenting extracts from the “joint” (e.g., Arksey, 1996; Polak and Green, 2015), or “couple interview” (Bjørnholt and Farstad, 2012) conducted with the founders (in date 22/05/2019), and the single interview conducted with the main teacher of the Milanese branch (in date 16/12/2018).

A residual part of the data presented and discussed in this paper are collected through what Wacquant (e.g., 2004; 2015) has famously labeled “observant participation,” namely the attempt to explore specific relationships, practices, and social processes as unfolding in social actors' “natural habitat,” thus avoiding the danger of gathering “dramatized and highly codified (re)presentation” (Wacquant, 2004:6) of the phenomenon studied. A presupposition of Wacquant's observant participation is his reliance on Bourdieu's concept of habitus, as both a “topic and tool” (e.g., Wacquant, 2004, 2013). Habitus, is here used as a topic of investigation in itself as it allows to inquire into: first, how Odaka Yoga's founders and practitioners acquire, transmit and modify the specific habitus that characterizes the ethos of their school; second, how the relationships between their habitus and the field in which they are positioned can be accounted for, that is, the homology between practitioners' positioning in the martial arts field and the broader sociocultural field; and third, the permeability and circular relationship between discourses (about health and self-transformation) and pedagogical practices. Habitus is also used as a research tool, that is, a privileged instrument “to decipher action and meaning from the body and with the body” (Sánchez-García and Spencer, 2013:5). More specifically, the use of habitus, which allows the researcher to approach the “social nature of [the] epistemic, affective, and moral dimension of embodied practice” (Sánchez-García and Spencer, 2013:1) as a tool, is pivotal to the observant participation of the apprenticeship process. Here, “the apprenticeship of the researcher can be adopted as a mirror of the apprenticeship undergone by the empirical subjects of the study” (Wacquant, 2011:81).

Summarizing, these methodological sketches of the multimodal approach and my reading of Jennings' theory of martial creation, share a commitment to inquire into the processes of constitution of a specific martial activity, taking into account its discursive references, practitioner's narratives, and biographies and processes of apprenticeship. Moreover, it is also important to mention that throughout the data collection and writing phases of this paper I followed a “situated ethics approach” (Roulet et al., 2017), that is, I tackled the ethical considerations related to research praxis as an ongoing social practice highly shaped by contextual factors rather than by universal codes of conduct (Nyberg, 2008). More specifically, at the outset of my research in the Spring of 2018 I was introduced to the Milanese branch of Odaka Yoga by Beatrice (studio owner and senior Odaka Yoga teacher) as a “researcher-practitioner.” Naturally, this overt positioning influenced my positionality within the field in a number of ways, also partly determining the relationships and interactions that I had a chance to develop, attend to and witness during these nearly 3 years of immersion into the field. Most notably, this overt positioning granted an exemplary level of transparency and a balanced power relation between the researcher and the participants. For instance, all the participants interviewed were informed beforehand that our conversations would be used for academic publications; were asked if they preferred their voices to be represented anonymously or otherwise; and were reassured that I would share with them an advanced pre-publication manuscript draft in order to receive their feedbacks, comments, and corrections on my interpretation and analysis of what we discussed together during our interviews. Roberto, Francesca and Beatrice consented to the publication of a number of extracts taken from our interviews and agreed to be openly named in my work. Moreover, as previously agreed, I contacted them in date 12/08/2020 to notify that I was about to submit for publication a manuscript whose empirics were largely based on our interviews and practice together (this paper) and required their feedbacks, comments and corrections to the manuscript. Tellingly, Francesca and Roberto replied positively, stating that “we really like what you wrote, important work and well-executed” (personal communication in date 02/09/2020); while Beatrice suggested that I could possibly participate in a formal Odaka Yoga teacher training to discuss the findings of my work with the broader community of training teachers. As a final note, I also further agreed with Francesca, Roberto and Beatrice that I will send them the final version of the manuscript, once published.

The remaining of the paper is dedicated to a dissection of Odaka Yoga and its conception of health, starting with a discussion of the birth of this style as recalled through its founders' biographies.

## Blending Martial Arts and Yoga for Health

### The Birth of Odaka Yoga

Odaka Yoga is a relatively recent style of postural yoga blended with martial arts elements. It was founded in Rome by the Italian teachers and entrepreneurs Roberto Milletti (also known as *Sensei*^5^) and Francesca Cassia (*Niji*^6^), in 1993. Roberto and Francesca do not only teach and run their business together, but they are also a life couple, as it is often the case in the martial arts field (e.g., Jennings, 2016). The official website of the school introduces Odaka Yoga as:

an innovative style of yoga with over thirty years of experimental research. Roberto Milletti and Francesca Cassia give life to a new concept: “Live Centered, Liquefy Your Limits, Embrace The Power,” which finds inspiration by observing the movement of the ocean and its waves. This is where in bringing together the idea of Bushido (the way of the warrior), zen and yoga, the principles of transformation, adaptability and interior strength are expressed physically and emotionally.

In other words, the philosophical and practical backbones of Odaka Yoga are predominantly concerned with a strategic rendition of a variety of Asian practices and traditions that are seen as vital to the practitioner's ability to embody and reproduce a centered, limitless, and empowered life through principles of transformation, adaptability, and interior strength. As such, Odaka Yoga can be mainly understood as a particular expression of the individualizing, self-transforming, and self-actualizing ethos of contemporary societies and the progressive role that the commodification of Asian spiritual and religious resources play in shaping these socio-cultural processes (e.g., Carrette and King, 2005; Altglas, 2014; Banesch, 2014; Jain, 2014, 2020). I will come back to this analysis, but for the moment, it is important to underline that Odaka Yoga is the outcome of decades of personal practice, as well as professional engagement and experimentation at the intersection of the martial arts, fitness, and yoga. In the following fragment, Roberto recalls his progressive involvement with martial arts and yoga:

I started very young with martial arts. At the age of seven. I approached martial arts because I was, in between brackets, a little bit bullied, at school, in the courtyard and so I was looking for self-confidence, I was trying to win my fears, my shyness. So, my grandpa suggested me martial arts, and from there, in short, all my path. Martial arts became an integral part of my existence, and then I met yoga when I was twelve, thirteen years old. And so, two great loves that I cultivated until they merged one with the other. So martial arts elements with the traditional poses of yoga, and then the meeting with Francesca, that comes, in short, from the world of fitness.

Francesca is the co-founder of Odaka Yoga. As Roberto, she is one of the 14th Yoga Alliance International Australia Master Yoga Platinum, the highest recognition issued by the yoga industry^7^. Moreover, Francesca has recently become the chief editor of “the world's largest and most influential yoga brand”^8^ the Yoga Alliance International online Journal (see text footnote 7, respectively). As she summons up:

I take a slightly different route. I am the generation of Jane Fonda. I did not have any intention whatsoever to do yoga. I love movement, I come from aerobics and suits, you know those eighties style, in lurex and when I was studying at university I also started to teach aerobics and then there was this gym in Rome where they were doing some yoga courses, and my sister who was studying psychology told me: “Look, let's go, I want you to have this experience,” [she said it] once, twice, three times, and in the end I said: “Ok, let's go.” It has been really like a revelation for me. From there on I have never abandoned the yoga mat. This incredible contact with the body, that was not only a tool for movement but that really talked to you, it was really like, oh yeah, I found my way, so to say. I found what I wanted to do in the world. And with Roberto we have, I love anatomy, all that more structured part that concerns movement that married very well with Roby's [an affectionate term for Roberto] creativity and this fusion with martial arts and yoga. And so slowly, slowly everything gained structure.

As Francesca approached the practice of yoga she was struggling with anorexia. Luckily, thanks to her dedication to the practice and “the physical realization of certain things,” as she says in our interview, she managed “to overcome it.”

Simplifying, Odaka Yoga is then the expression of Roberto's and Francesca's respective experience and proficiency with martial arts and fitness. More precisely, it is the outcome of the merging and complementing of their different expertise in the development of an innovative style of postural yoga characterized by a simultaneous reliance on Asian and Western systems of knowledge. However, when Roberto and Francesca met and began their lifelong journey together, Odaka Yoga, as we know and practice it today, was not yet formalized. It was only in 1993 that the word “Odaka,” as much as Odaka Yoga's teachings, were subjected to copyright. However, “Odaka” was already used as an acronym of the martial art and yoga studio founded in Rome and run by Roberto since 1984 (later also in partnership with Francesca) which was called Oriental Disciplines Arashi Kyo Academy (O.D.A.K.A). As Francesca further explains:

Arashi Kyo is the name of Sensei, of Roby, when he was European champion of Karate. I mean [we taught] many styles but the acronym adopted was Odaka, and so everyone [said]: “I am going to Odaka,” “I am going to Odaka,” “Odaka,” “Odaka.”

O.D.A.K.A functioned as a hub, a place for practical experimentation and philosophical discussion over the meaning and purpose of martial arts. Its main goal was the diffusion of specific disciplines as means of inner knowledge and spiritual search to favor the perfect mind and body harmony development of each individual^9^ Most Notably, Roberto was teaching two martial arts systems he had created: *Shin Jitsu Ryu* and *Wi Yoga Wakan*. These martial systems emerged at the intersection of Roberto's experience and experimentation with Karate Shotokan, Ju Jitsu, zen, Hatha and Raja yoga and offered already the philosophical and practical bases for what later on would develop and evolve as Odaka Yoga. Of course, Francesca was also instrumental to the development of Odaka Yoga through her extensive experience in the world of fitness and yoga and her martial arts apprenticeship under Roberto's guidance.

Today, Roberto and Francesca travel the world to share their hybrid style of yoga and are considered among the most influential contemporary teachers. For instance, Roberto has also been featured in ‘*Om Yoga Magazine UK*’ as one of the three world leaders in new, contemporary forms of yoga^10^. Their style has evolved into a transnational organization with centers across four different continents and a hectic calendar of events such as festivals, workshops, and teacher trainings that take place continuously all over the world and whose online diffusion has been further fostered by the current Covid-19 pandemic. Summarizing, Odaka Yoga is constituted by two different but parallel pillars: one is the discursive and practical reference to Asian systems of knowledge such as yoga, zen, and martial arts, whose main influence comes from Roberto's experience, experimentations and creativity; the other, is a focus on Western science and medicine, and more specifically on anatomy and biomechanics, primarily inspired by Francesca's background in the fitness world.

Odaka Yoga further developed internationally because of a serendipitous happening, or more specifically an epiphany, that brought Roberto and Francesca to drastically re-imagine their life. After their martial academy shook due to internal conflicts, its key members took different routes, and Roberto and Francesca moved to Australia where they were planning to open a new studio and continue their work of dissemination and martial explorations. As discussed by Francesca, watching the ‘*The Last Samurai*’ (2003)^11^. at the cinema represented a turning point in their life:

Everything started because 1 day we were living in Australia, in a beautiful place where we practiced with joy, and “The Last Samurai” came out in the cinema, and we went to watch it…we were in Melbourne and we had just rented a beautiful place for a new yoga studio. We left [the cinema] and Roby did not talk for a couple of days. I said to him “What's happening? How are you feeling?” He replied, “Look, I touched the soul, deeply, and I feel I need to go to Japan.” And in that moment, I thought “Give him a couple of days and he will be over it.” But it did not happen. Instead we terminated the contract [of the yoga studio] and we sold everything we had in Australia [where] we were only a step away from the permanent visa. There was our best friend that was saying “I mean, you don't speak a word in Japanese, you know nobody, but where do you want to go?” But instead his soul had launched this message, such an intense message that we moved there. The first year, the first few months in Japan were rather difficult, but then the local community begun to open up.

Watching ‘*The Last Samurai*’ prompted Roberto to silently question his current life and ongoing projects, further challenging his long-standing, and ever evolving “martial habitus” (Brown and Jennings, 2013) which, in all fairness, was already shaken by and re-adapting to the recent disruption of his academy. Because of these crises Roberto and Francesca re-located to Japan, where they creatively managed to further innovate their martial style and finally obtained international recognition. Recalling Jennings' (2019:69) six stages of his theory of martial creation in the light of the biographical remarks here discussed it is possible to provide the following analysis of the birth and development of Odaka Yoga:

1. Roberto and Francesca have a background as *practitioners* in one or more martial art(s). More precisely Roberto practiced Karate Shotokan and Ju Jitsu (in both of which he hold a Fourth Dan Black Belt) next to zen, Hatha and Raja yoga; while Francesca practiced Karate (earning a First Dan Black Belt under Roberto's tutelage) next to a lifelong practice with different styles of yoga and fitness.
2. Both Roberto and Francesca reached a level of *competence, confidence*, and *charisma* that did not only allow them to gather a following, but also to transform a local martial arts academy into a transnational organization praised for its innovative character by some of the most influential voices of the contemporary yoga industry (e.g., Yoga Alliance).
3. Roberto and Francesca were *not* the official gatekeepers or lineage holders of their original system (e.g., Karate Shotokan, Ju Jitsu, Hatha or Raja Yoga). However, Roberto was the founder of two different martial arts (e.g., Shin Jitsu Ryu and Wi Yoga Wakan) that in due time, and with the contribution of Francesca, morphed into Odaka Yoga.
4. Roberto and Francesca started to practice martial arts and yoga as an answer to a personal *crisis* that aggravated them. More specifically, when still a child Roberto was bullied and attempted to exorcize his fears through the martial arts, as suggested by his grandfather; while Francesca, already familiar with the fitness world as she was teaching aerobics as a side job to her study in business administration, found in yoga an effective tool to cope with and resolve her anorexia. A Further moment of crisis was represented by the disruption of their Oriental Disciplines Academy in Rome, which brought the two teachers to Australia where they continued their martial explorations. Finally, Roberto's conversion following the vision of “*The Last Samurai*” functioned as an epiphany that proved instrumental in the further internationalization and popularization of Odaka Yoga.
5. Roberto and Francesca, each of them through their own expertise, devised a *solution* for their personal problems through a revised martial and human development system, in this case Odaka Yoga. More specifically, through the formulation of their “new concept,” “Live Centered, Liquefy Your Limits, Embrace The Power” and its “principles of transformation, adaptability and interior strength,” Roberto and Francesca provided an existential answer to the fears and health challenges encountered in their youth while also offering a seductive path cable to equip its practitioners to face the flexibility demands, performative requests, and uncertainties of contemporary neoliberal societies.
6. Although Roberto and Francesca are still alive and well, there are preliminary elements to postulate that their death will eventually create added *chaos*, thus fuelling the cycle of *creativity* among future generations of practitioners and possible lineage holders, a fascinating topic in itself that here cannot be competently explored.

As Jennings' (2019:66, emphasis in original) comments, these six stages can be summarized in the following declaration: “*A martial art is founded by a disciplined, habitual martial artist who creatively transcends personal and social crises.”* Here, Roberto and Francesca learned from their teachers (of whom unfortunately I know very little if not nothing), practiced extensively and creatively, achieved a status within their respective fields and finally were able “to create something new and create a new method of achieving it” (Jennings, 2019:66). As testified by the biographical sketches here provided “[t]he overall process of creativity is a potentially lifelong process, but it also comes with fleeting and intensive moments of (sometimes epiphanous) creation” (ibidem).

Enriching Jennings' theory of martial creation with a closer look at Roberto's and Francesca's habitus and positions within the martial arts, yoga, and fitness fields, may further help focusing on important elements in the birth and development of Odaka Yoga. First, Roberto and Francesca managed to adapt and transform their respective “martial” (Brown and Jennings, 2013) and “fitness” habitus (Sassatelli, 2002) into a specific declination of “yogic habitus” (Di Placido, 2018) hybridized with martial arts and fitness elements. This signifies that their old-habitus and its schemes of dispositions, developed at the intersection of the martial arts, fitness, and yoga fields, progressively changed as they moved away from the center of the martial arts and fitness fields, and toward the center of the yoga field. In other words, we may say that Roberto and Francesca successfully managed to exploit and convert certain types of capitals that granted them their previous positioning in the martial arts and fitness fields, into useful resources to acquire an ever more central positioning within the yoga field. For instance, next to their previous teaching expertise and pedagogical know-how—including in the martial arts founded by Roberto, yoga, and aerobics—they exploited the “bodily” (Wacquant, 1995) or “physical capital” (Shilling, 1991, 2004, 2012) developed, acquired, and secured through their lifelong involvement with different martial activities and fitness practices, to build an image of successful, competent, and innovative teachers. In so doing, as they skillfully converted their proficiency in the martial arts and fitness worlds to create an innovative hybrid style of yoga, they also contributed to the reproduction of the relationship of homology between the broader field of power, and the martial arts and fitness field. Simplifying, via their capitals (e.g., economic, cultural, physical, and so on), Francesca and Roberto secured a dominant position within the martial arts and fitness fields, while also managing to remain in a dominant position in their successive and progressive involvement within the yoga field.

Finally, I would like to conclude this section with a short analysis of the manners in which Roberto's and Francesca's biographical remarks could be effectively accounted for through the lenses of Foucault's (1986, 2005) “epimeleia heautou.” First, Odaka Yoga functions as a moral framework that guides practitioners' conduct and teleological orientation in the world, more specifically through its concerns for practitioners' health and processes of self-transformation as filtered through the concept of “Live Centered, Liquefy Your Limits, Embrace The Power”; second, it offers the possibility to convert one's attention from the outside world toward one's own interiority, thus promoting a specific ethic of self-care, self-actualization, and self-transformation that is already well-established in Western renditions and interpretations of Asian religious and spiritual resources; and third, it offers an array of practices, or technologies of the self, through which practitioners are invited to cultivate, interiorize, and master the specific worldview advocated by the school and deepening further their self-transformative experiences of conversion in the light of the discourses and practices advocated by the founders. Interestingly enough, it is also as a consequence of a dedicated engagement with martial activities as care of the self, that Roberto and Francesca managed to successfully adapt and change their martial and fitness habitus, and progressively translate their capitals, skills, and expertise from the martial arts and fitness fields into the yoga field.

The next section is entirely dedicated to a discussion of Odaka Yoga's conception of health through an analysis of its understanding of the body as framed at the intersection of Asian and Western systems of knowledge.

### The Odaka Yoga Body: Between Medicine and Spirituality

Odaka Yoga presents an understanding of the human body framed at the intersection of Asian and Western systems of knowledge. In other words, the Odaka Yoga body is built on a hybrid conception of health, simultaneously inspired by biomedical/anatomical and esoteric/spiritual principles. In the following I provide two extracts that testify to this hybrid conception of health. The first field note was written right after a teacher training weekend held in Milan in the Winter of 2018. Here, Francesca warns me against hyper-flexing the legs and instructs me about the physical cues to notice in order to know if a movement is more damaging than benefiting:

We are going through a short sequence that involves the stretching of the leg, calf, tight, and quadricep. Each one practices individually, Francesca walks around, looking and giving suggestions. One knee on the floor and the other leg stretched in front, heel, and big toe on the floor. When I try to place the sole of the foot on the mat I feel a burning unease around the knee and on the back of it. I almost lose balance and my back stiffens up too. A perfect yogi! I then do the pose stretching the leg but touching the mat only with my heel and it goes better. As Francesca passes by, I tell her what I felt when I tried to bring the sole to the mat. She then looks at me and says: “You should never hyper-stretch anything. Especially if you feel the stretch behind the knee or at the end of the calf and of the tight, closer to the knee. If the warmth, the stretch is not at the center of the muscle then bend the knee. It means you are working beyond the capacity of your muscle and you are forcing it” (16/12/2018).

In the second field note, taken during a weekly class at JustB, the Milanese headquarter of Odaka Yoga where I undertook the greatest part of my research, Beatrice, Odaka Yoga teacher and studio owner, evokes the energetic constitution of practitioners' bodies:

After this intense, central part of the practice we move in a standing position. The transition is done from the dog bringing one leg in between the arms and then the other following it. Beatrice says: “Slowly unwind your back, vertebra after vertebra, the arms opening, as large as you can. The palms meet over the head and come down slowly with the elbow open and the finger well-spread. Open again. The hands meet and come down, this time the wrists rotate and the arms are again open, with the elbow at ninety degrees and the palms open.” We repeat this and similar movements a few times [this movement is known as ki flow by Odaka Yoga practitioners] and then we move on to the performance of a warrior sequence. At the end of this sequence while we are again standing at the beginning of the mat, Beatrice says: “Now place your hands a few fingers below your belly button. Here is the tanden, the center. All our own energy comes from this energy center, all movement comes from here. It is known in all Oriental traditions as the most important energy center (31/05/2018).

The first fragment depicts Francesca's attention for the technical aspects of the practice and more specifically for a practice oriented toward anatomical details and injuries prevention. The body that emerges from this type of attention is substantially a biomechanical and medicalized body. The Odaka Yoga's website seems to further reproduce this vision:

Odaka Yoga re-educates the body to move in its entirety by reawakening the intelligence of our bodies. Specific sequences are created for different areas of the body while working on postural alignment in order to prevent muscle, skeletal, and tissue injury. During an Odaka Yoga session, muscles are activated with balanced and functional movement that simultaneously strengthen and elongate^12^.

Biomechanics, as advocated by Odaka Yoga, is the study of the structure and functioning of the human body in motion. Its two main aims are to enhance performances and prevent injuries. Biomechanics implies a specific idea of the body: an object to be closely scrutinized for the discovery of its most functional (read natural and effective) movement patterns. The result of this reification is that the body, seen through the lenses of biomechanics, becomes a “medicalized body” (Robbins, 2018), understood as that specific body amenable to scientific scrutiny and thus exposed to a certain “medical gaze” (Foucault, 1973).

As Foucault has poignantly shown, between the end of the eighteenth century and then throughout the nineteenth century, the body was substantially re-casted as an object of knowledge at the intersection of science and medicine. From this perspective, biomechanics can then simply be considered as a later development of the epistemic transformation traced by Foucault in his genealogical study of the emergence of modern medicine. The medicalized body is then dissected by the medical gaze which penetrates its secrets, uncovers the mysteries of its functioning and translates them into a visible system of representation where bones, muscles, tendons, and organs are simultaneously: (a) seen as self-standing objects; (b) seen as the parts that form the medicalized body. The pedagogical consequences of this understanding of the body as much as its influences in shaping a specific conception of health based on anatomy and Western medicine, are among the most obvious implications of the Odaka Yoga body as a medicalized body. In fact, the primary consequence of this displayable body is that through the lenses of disciplines such as biomechanics, the body is potentially detached from its experiential dimension to acquire instead a more visual, object-like, anatomical identity (Foucault, 1973; Robbins, 2018). This is precisely what grants the suspension of the body as the locus of personal experience, and promotes the legitimization of different areas of expertise to emerge, where doctors, scientists, couches, and yoga teachers become the qualified holders of the hidden truths of the body. Here, next to “re-education,” “alignment” and “protection from injuries,” Odaka Yoga wants to cultivate, this time through the “activation,” “balancing,” “strengthening” and “elongation” of the muscular band, a very specific declination of the medicalized body^13^.

On the contrary, in the second field note proposed at the beginning of this section, Beatrice depicts a portrait of practitioners' bodies as primarily grounded in their energetic constitution. Here, the “liquid style approach” of Odaka Yoga, obtained by merging the martial arts flow of inner energy, and the zen spirit of quieting the mind with yoga postures, produces a practice akin to “an unremitting motion, a wave-like movement, a process where no interruption occurs between one pose and another.” This type of fluid movement “aims to dissolve physical, mental and emotional tensions, while soothing the mind with immediate action”^14^. More specifically:

Once the restraint is transformed, our habitual pattern of movement and thought is dissolved and each position creates space for a dialogue between our deepest fears and the possibility of overcoming them. The Odaka flow allows for us to go deep within ourselves and become “liquid.” Odaka Yoga allows you to embrace adaptability while keeping an inner peace. It becomes a complete fluid and transformative body-mind experience….and one you will not forget^15^.

At the heart of this conception of the body there is the “subtle body” model, that specific understanding of the body where its energetic structure interacts with and affects the anatomical or physical body of the practitioner (Johnston and Barcan, 2006). This model, first popularized in the West in the nineteenth century by the esoteric writings of the Theosophical Society, has its origins in the ‘*Upanishads*’, a collection of Sanskrit sacred texts, more specifically the ‘*Brhadaranyaka Upanishad*’ (IX–VI B.C.E), the ‘*Katha Upanishad*’ (V B.C.E) and the ‘*Taittiriya Upanishad*’ (V–VI B.C.E) (Samuel and Johnston, 2013; Mallinson and Singleton, 2017). The ‘*Taittiriya Upanishad*’ describes a subtle body model composed of five sheaths (*koshas*). These koshas are, in the context of yoga soteriology, a series of interconnected layers that range from gross energy (matter) to progressively more refined subtle qualities, the subtlest of which are the states of dreamless sleep and liberation (*samadhi*). They are, from the grossest to the finest: *anna-maya-kosha* (physical body), *prana-maya-kosha* (energy body), *mano-maya-kosha* (mind body), *vijñana-maya-kosha* (consciousness body), and finally *ananda-maya-kosha* (bliss body) (Samuel and Johnston, 2013:33).

The subtle body model presented in the Upanishads, and later re-elaborated by the yogic, tantric and other Asian traditions, is constituted by a series of focal points (*chakras*) connected by specific conduits or channels (*nadis*) through which the subtle breath (*prana*) moves. According to this subtle body model, the flow of prana–within and across these sheaths–determines not only the physical and mental state of individuals, that is, their health, but also the plausible acquisition of supernatural powers, immortality or liberation (White, 2009). In other words, according to this theory, when yoga practitioners perform physical postures (*asana*) or breathing exercises (*pranayama*), among other practices, they are not merely engaging or manipulating the physical body, but also the (ephemeral) energetic substrate of their being. As Johnston and Barcan (2006:30) rightly underline:

[i]ntervention in one [sheaths] of necessity means intervention in others. The dominant belief is that changes in this energy (in this subtle body) at any level—mentally, physically, emotionally, spiritually—will bring about changes to all other aspects of the individual.

This is exactly the theory behind Odaka Yoga's reliance on the subtle body model as an integral part of its hybrid conception of health. From this it follows that Odaka Yoga's transformative potentials are entirely based on the healing power of prana as it freely circulates across the different layers of practitioners' subtle bodies, “a union that aims to dissolve physical, mental and emotional tensions” through the “flow of inner energy.”

At this point, it is important to note that the subtle body of Odaka Yoga is not purely derived from the yoga tradition just discussed. On the contrary, it presents two main, although interconnected, differences: first, it downplays the importance of the breath as a conduit for prana emphasizing instead the importance of fluid movements; second, it ascribes specific importance to the energetic reservoir known in many Asian martial arts as *tanden* (or *dantian*). In relation to the former, Odaka Yoga subdues yoga's traditional emphasis on the breath to the importance of fluid movements. Here, the “liquid style approach” is obtained by merging (a) martial arts elements, (b) a focus on the inner flow of energies, and (c) the emulation of the yielding nature of the Ocean waves with yoga postures. In fact, it is through this liquid style and its fluid movements that the energy circulates across the different koshas, allowing practitioners to transform their physical, energetic, mental, emotional, and even spiritual makeup. In relation to the latter, Odaka Yoga reframes yoga's traditional subtle body model focused on chakras through the idiom of Asian martial arts. Here, the tanden, or more specifically the lower tanden^16^ (hereafter simply tanden), becomes the most prominent energy center of the practitioner's body. The tanden corresponds to the first two chakras of yoga' subtle anatomy (*muladhara* and *svadhistana*). According to Asian martial arts it indicates the reservoir of one's own vital energy, what in the yoga tradition is usually known as *kundalini*, a dormant snake coiled in the muladhara chakra whose stimulation, awakening and vertical rising across the other six main chakras is seen as bearer of supernatural powers, longevity and/or enlightenment (Eliade, 1973). Partly following this model, the tanden is simultaneously understood as an energetic and spiritual center: it is not only the main source of *ki* (the Japanese equivalent of prana) but the center of gravity of the body itself, the center from which all movements spring from as previously evoked by Beatrice during her class.

Nonetheless, to return to my main argument, the most important aspect of this model, whether we see it as belonging to the traditional model introduced in the ‘*Upanishads*’, or a later re-interpretation imported from Chinese medicine or the martial arts, is that it displays a model of the body that is substantially antithetical to the anatomical and biomechanical conception of the medicalized body previously discussed. This signifies that if the medicalized body—as a whole or in its parts—responds to a meta-narrative where scientific and medical discourses are woven together making the body an object to be closely scrutinized by a plethora of experts; the subtle body is instead regulated by the flows and functioning of energy, which is itself comprised—according to yogic philosophy—by the constant interplay between the grosser and the finest koshas. This specific understanding of the body, then, gives rise to a very particular conception “…of embodied subjectivity in which matter and consciousness are not understood as ontologically distinct but as varieties of ‘energy’ resonating at different densities” (Johnston and Barcan, 2006:25). Here, the medical gaze described by Foucault loses all its representational and explanatory power and the truth of the body returns to become a mystery which science and modern medicine cannot competently understand just yet. Moreover, where the medical gaze fails to bring into light the invisible, practitioners' direct experiences of this invisible flow of energy, as the following field note demonstrates, become the primary epistemological ground on which the subtle body model can be understood and made sense of. Here, I discuss the sensuous and emotional dimensions of being able to perceive one's own inner flow of energy at the end of an Odaka Yoga practice:

The emotions and the energetic sensations that the practice and the final relaxation induced in me are exhilarating. I feel like walking to Beatrice to tell her that every single lesson I do with her I feel like crying. But I stay put, sitting on my mat, while everybody else is slowly getting up, and going to the changing room. When the traffic is somehow slowing down, I also stand up and go to take the spray and the tissues to clean up my mat. As I walk toward the corner where the cleaning supplies are, literally a few meters away from my mat, I feel my body is light, relaxed, open. I feel happy. I really feel a great amount of vibrations in my belly area, especially in the upper belly part, and flows of relaxed energy all over my limbs (31/05/2018).

At a practical-discursive level the body proposed by Odaka Yoga is simultaneously: (a) a medicalized body understood through the lenses of anatomy and biomechanics; (b) and a subtle body, framed according to traditional yogic and martial arts subtle anatomy. This tension is well-exemplified in the following fragment extracted from an interview with Beatrice:

With this practice modality that is fluid but not too fast we work exactly on prana, on the energetic quality. It is an energetic quality where you are not exhausted, it is tiresome, but never extremely tiresome. You sweat but not too much. The body is always thermoregulated and is never over heated. Also, when you sweat a lot, you actually sweat a lot because outside is really hot, so you are self-regulating. [This style] creates a sort of energetic charge. In this way we work a lot on the connective tissue. And [to work] on the connective tissue means working on the emotions, because it is in the connective tissue that the strongest emotions pass through.

Beatrice's narrative underlines how, according to the subtle body model, the “liquid style approach” of Odaka Yoga and its primary focus on energy work, is inextricably connected with a manipulation of the connective tissue of practitioners' bodies, that in turn is linked to practitioners' emotions. We can then clearly see how this model, thanks to the constant interplay across sheaths, offers the right philosophical and discursive foundations to frame a practice where energy work, physical work, and emotional work, are not only manifestly overlapping on an epistemological level, but are also experientially accessible to the practitioners. Yet, what is perhaps of greater interest is that this interconnectivity among sheaths is here reframed through the language of modern medicine, and science, with terms such as “thermoregulates,” “self-regulating,” and “connective tissue,” making it almost impossible to disjoint these two registers. The medicalized and the subtle body are no longer recognizable as separate bodies. Discourses belonging to these two different registers are then simultaneously mobilized to reciprocally legitimize one another, and strengthen their respective therapeutic, self-actualizing and self-transformative framings of the body.

In the next section I provide a brief discussion of how Odaka Yoga' conception of the body is epitomized, reproduced, and embodied by its ideal-typical practitioner, the Odaka Yoga Warrior.

#### The Odaka Yoga Warrior

The Odaka Yoga Warrior is the ideal-typical practitioner capable “to live centered in the middle of chaos^17^.” This signifies that the Odaka Yoga Warrior maintains her “center” and a “calm mind”; it seeks to “transform” herself cultivating “new balance” and “inner strength” while embracing the adversities of life and its “challenges.” For the Odaka Yoga Warrior:

Challenges should be thought of as opportunities, because that is exactly what they are. Without challenges, we cannot attain inner power. This makes challenges essential to growth. When we give into challenges without any fight, *we embrace the empowerment* (emphasis in original)^18^.

This ideal-typical practitioner emerges at the intersection of the practical-discursive logics of Western medicine (with its anatomical and biomechanical principles) and Asian systems of knowledge (and their fluid movement and inner flow of energy) next to their respective models of the body and conceptions of health. Moreover, the Odaka Yoga Warrior is simultaneously a product and a “solution” to the uncertainties, flexibility demands, and challenges of contemporary Western societies. Having extensively inquired into Odaka Yoga's conception of health in the previous pages I devote the remaining of this section to a discussion of the affinity between the Odaka Yoga Warrior and a neoliberal mode of thought. As the Odaka Yoga's website argues:

To become an Odaka Yoga warrior means to become adaptable, flexible, and fully integrated: a complete fluid and transformative body-mind entity. When moving in the Odaka flow, muscles are invited to engage in a new way which strengthens them and, in turn, creates movement in the mind. If the connective tissue is tense, muscles are no longer able to move freely because the entire body tenses and tightens, and so does our mind^19^.

I would like to underline how the Odaka Yoga Warrior is not merely the outcome of the meeting between the practical-discursive universes of Western medicine and Asian systems of knowledge but is also the expression of the particular ethos of contemporary neoliberal societies. In fact, “[t]o become an Odaka Yoga warrior means to become adaptable, flexible and fully integrated,” or in other words, “a complete fluid and transformative body-mind entity.” This depiction of the Odaka Yoga Warrior—as much as its elective affinities with challenges, growth and empowerment—I argue, substantially adheres to the normative biopolitical injunction of self-care, flexibility and self-responsibility that characterizes contemporary neoliberal societies across social domains, such as higher education, sport and the labor market (e.g., Andrews and Silk, 2012; Bélanger and Edwards, 2013; González-Calvo and Arias-Carballa, 2018; John and McDonald, 2020). Understood in this light, the Odaka Yoga Warrior is—thanks to her self-control, self-mastery, and constant work of self-cultivation—able to fruitfully merge the central tenets of the medicalized and subtle body models within a unified therapeutic framework of fluid self-care and self-actualization. If this analysis is correct, then the Odaka Yoga Warrior is a particular declination of the “neoliberal yogi” discussed by Godrej (2016), that is, a yoga practitioner whose conduct is substantially in alignment with the dominant paradigms of self-care, self-conduct and self-responsibility of contemporary neoliberal societies. Here, Foucault (2008) concept of “biopolitics,” defined as the control of the welfare, wealth, longevity, and health of the population through the pervasive government of individuals' conduct, becomes a useful heuristic tool to theorize about the interconnections between Odaka Yoga's discursive focus on heath and self-transformation; its practical repertoire oriented to the problematization of practitioners' health, its maintenance and cultivation; and the type of power—or in Foucault's terminology governmentality—that dominates our everyday conduct as much as the ethos of our contemporary societies. These reflections point to the fact that, as much as it is pivotal to understand martial artists' positioning within the broader field of power in order to fully understand the martial systems that they create, it is also important to understand the broader ethos of a field, of a social group or even of an entire culture, to fully account for its conceptions of health and the manners in which they may be discursively framed, reinforced, or in certain cases also disqualified.

## Conclusion

In this paper I have investigated the birth and development of Odaka Yoga and its conception of health. In order to do so I have relied on Jennings' theory of martial creation enriched by some of the central analytical tools of theorists such as Bourdieu and Foucault. Naturally, more should be done in this direction, for instance exploring Bourdieu's notion of habitus at a dispositional level, that is, at the level of its constitutive elements. This would allow to show how martial artists' habitus resists specific biographical or sociocultural transformations or on the contrary evolves and drastically changes following personal or social crises. Similarly, more should be said in relation to martial artists' positions of homology in the field of power and in the martial arts field, for instance focusing on the cultivation, acquisition, and conversion of specific types of capital, further exploring the manners in which martial artists successfully mobilize pre-existing capitals to establish their new martial styles.

Nevertheless, the central argument proposed in the paper is that Odaka Yoga's conception of health derives from a framing of its practitioners' bodies at the intersection of Western and Asian systems of knowledge, giving shape to a body that is simultaneously medicalized and subtle or energetic. It is here that the Odaka Yoga Warrior emerges, the ideal typical practitioner whose defining traits are its strength, adaptability, flexibility and integration of mind, body, and spirit. Also as briefly mentioned in the previous section, the focus on practitioners' health and processes of self-transformation is not merely a trait of Odaka Yoga, but one of the surfaces in which the normative injunction of contemporary neoliberal societies are expressed and socially reproduced, in practice, at a meso and micro level.

I would like to conclude this paper with a reflection on hybrid conceptions of health and the ubiquitous role of health discourses and narratives across sociocultural domains. Two examples should suffice: the psychologization of religion and spirituality and the spiritualization of medicine. The former represents one of the most tangible trends in the contemporary religious and spiritual fields, that is, the adaptation, evolution, and development of specific messages of salvation within the context of a pervasive “therapeutic culture” (e.g., Furedi, 2004; Illouz, 2008). As rightly underlined by Altglas (2014) religious and spiritual resources are increasingly re-casted in the language of emotionalism, self-help and psychologism, to the point that self-transcendence, one of the traditional pitfalls of religious and spiritual paths, has progressively morphed into a search for self-actualization, one of the landmarks of consumer popular culture. Here, religious and spiritual resources “are understood as the self-help tools helping to acquire detachment, strength, optimism, and acceptance, which as such pave the way to self-realization and self-fulfillment” (Altglas, 2014:216), as the case study of Odaka Yoga and of its ideal-typical practitioner poignantly testify.

The latter, namely the spiritualization of medicine can be identified in the growing scientific, medical, and professional interests for complementary and alternative medicine (CAM) (e.g., Balboni and Peteet, 2017; Brosnan et al., 2018; Timmins and Caldeira, 2019), the expansion of what David Heelas has famously labeled the “holistic milieu” (Heelas, 2008), and the forms of knowledge production and social transformations that they foster. CAM and the holistic milieu are in fact characterized by the attempt to bridge Western biomedical conceptions of health with non-Western traditional medicine, such as acupuncture, Ayurveda and traditional Chinese medicine alongside other approaches such as Reiki, homeopathy and hypnotherapy. Here, practitioners are promised balance, relaxation and—perhaps most importantly—the restoration of their body-mind-spirit unity, through a dedication to psychosomatic techniques such as yoga, massages, and meditations of various kinds. Most notably, what defines CAM and the holistic milieu is that their effectivity and transformative potentials are granted by their scientific and medical framings, and of course oftentimes, by the experiential and ritualistic rendition of these disciplines and sociocultural domains. What I meant to underline through these two short examples is that although it is correct to claim that anxieties over health, its minute control and mastery are a defining feature of our contemporary societies and their form of governmentality, our sociological inquiries would benefit from a more detailed understanding of what types of health is at stake in different sociocultural domains, especially in those domains articulated around hybrid conceptions of health.

Finally, I contend that to study hybrid conceptions of health where Western, indigenous, and New Age systems of knowledge and their epistemologies intersect, could be a promising avenue for future research and may contribute to shed further insights into the psychologization of the religious and spiritual fields as much as the spiritualization of medicine. The concept of “hybrid field” (Pedrini et al., 2019) is, I believe, particularly useful in this regard. This concept was originally coined to discuss—starting from a micropolitical exploration of the coaching environment—how “boxe popolare”'s (people's boxing) (Pedrini, 2018, 2020) coaches negotiate the boundaries of the field relying on the practical-discursive logics of different fields, such as the sporting and the political fields. Its heuristic potential, however, could also be used to theorize about: first, how contemporary conceptions of health in the field of martial arts, for instance, derive from the meeting of different instances originating in disparate location of the martial arts field; and second, how they are imported from outside their boundaries (e.g., the medical field, the therapeutic field, the religious field, and so on). In the case of Odaka Yoga, I have preliminarily shown how its conception of health is strongly derived from the anatomical and biomechanical understanding of the body, inspired by Western medicine and its objectifying gaze, and the subtle body model of Asian traditions such as yoga and martial arts. This analysis, I believe, has helped defying any essentialist reading of Odaka Yoga as intrinsically commodified and devoid of substantive philosophical foundations, and in doing so, has also shown that Odaka Yoga is as much the expression of the self-centered and self-actualizing ethos of contemporary societies, as it is the outcome of decades of martial explorations at the intersection of yoga and martial arts.

## Data Availability Statement

The data used for this paper are: extracts of biographical interviews, ethnographic field notes and extracts from the Odaka Yoga's website. There is no restriction on the data. The first two types of data are integrally, and privately stored by the author. The thrid type is freely available online. Requests to access the datasets should be directed to Matteo Di Placido, m.diplacido@campus.unimib.it.

## Ethics Statement

The patients/participants provided their written informed consent to participate in this study. Written informed consent was obtained from the individual(s) for the publication of any potentially identifiable images or data included in this article.

## Author Contributions

The author acknowledges to be the sole contributor of the paper and to have collected all the original material here presented and conducted the analysis individually. No other authors were directly involved in the data gathering, the analysis phase and the writing of this paper.

## Conflict of Interest

The author declares that the research was conducted in the absence of any commercial or financial relationships that could be construed as a potential conflict of interest.

## Footnotes

^1^https://www.frontiersin.org/research-topics/12183/martial-arts-health-and-society (accessed in date 28/07/2020).

^2^Literally translated as “path/moral of the warrior”. It is the code of conduct of the samurai, the Japanese warrior cast.

^3^Jennings proposes the concept of habit(us) in the attempt to compromise between the pragmatist notion of habit and the classical sociological concept of habitus.

^4^“By *episteme*, we mean, in fact, the total set of relations that unite, at a given period, the discursive practices that give rise to epistemological figures, sciences, and possibly formalized systems…The episteme is not a form of knowledge (*connaissance*) or type of rationality which, crossing the boundaries of the most varied sciences, manifests the sovereign unity of a subject, a spirit, or a period; it is the totality of relations that can be discovered, for a given period, between the sciences when one analyses them at the level of discursive regularities” (Foucault (1972:191, emphasis in original).

^5^Sensei is a Japanese word often translated as ‘master’ or ‘teacher’, the corresponding of the Sanskrit guru. The term sensei does not refer to somebody that transmits mere technical knowledge but to an individual imbued with actual wisdom.

^6^Niji is an archaic Chinese term that addresses the recognition of time, or better the awareness that time has come for a specific action to be undertaken.

^7^https://odakayoga.com/en/francesca-cassia/ (accessed in date 14/02/2020).

^8^https://www.yogajournal.com/page/about-us (accessed in date 06/10/2018).

^9^http://web.tiscali.it/odaka/odaka.htm (accessed in date 08/08/2020).

^10^https://odakayoga.com/en/odaka/roberto-milletti/ (accessed in date 05/08/2020).

^11^In a word, ‘*The Last Samurai*’ narrates the story of Nathan Algren (Tom Cruise) an ex-American soldier in charge of the training of the Japanese Imperial army. Eventually Nathan Algren, captured in battle by the rebellious samurai, joins their cause in a romantic and deadly confrontation over two ways of life and ethics of war.

^12^https://odakayoga.com/en/ (accessed in date 07/02/2020).

^13^Interview conducted with Francesca Cassia and Roberto Milletti (in date 22/05/2019).

^14^https://odakayoga.com/en/about/concept/ (accessed in date 10/02/2020).

^15^https://odakayoga.com/en/what-is-odaka-yoga/ (accessed in date 04/08/2020).

^16^Especially in the context of Japanese martial arts the tanden is closely related to another important energetic/anatomical center positioned in the lower abdomen, the *hara*, where the samurai believed their spirit resided. It is important to notice how the tanden and the hara closely resemble the sheaths model previously discussed as they simultaneously function as a point of intersection between the physical body, one's own psychological and emotional constitution, and the qualities of one's own inner energies. According to a variety of martial arts there are three to five tanden. The first and most important is the lower tanden discussed in the text. The median tanden is located in the upper chest area in the heart region and roughly corresponds – in positioning and functions – to the fourth chackra, *anahata*. The higher tanden is in turn located in between the eyebrows and corresponds to the sixth chakra *ajna*. For a more in-depth discussion of the main chakras and their functions see Samuel and Johnston (2013).

^17^https://odakayoga.com/en/ (accessed in date 07/02/2020).

^18^https://odakayoga.com/en/in-defence-the-way-of-the-urban-warrior-2/ (accessed in date 10/08/2020).

^19^https://odakayoga.com/en/about/concept/ (accessed in date 13/02/2020).
